# Cleaved Form of Osteopontin in Urine as a Clinical Marker of Lupus Nephritis

**DOI:** 10.1371/journal.pone.0167141

**Published:** 2016-12-19

**Authors:** Koji Kitagori, Hajime Yoshifuji, Takuma Oku, Chiyomi Sasaki, Hitomi Miyata, Keita P. Mori, Toshiki Nakajima, Koichiro Ohmura, Daisuke Kawabata, Naoichiro Yukawa, Yoshitaka Imura, Kosaku Murakami, Ran Nakashima, Takashi Usui, Takao Fujii, Kaoru Sakai, Motoko Yanagita, Yoshitaka Hirayama, Tsuneyo Mimori

**Affiliations:** 1 Department of Rheumatology and Clinical Immunology, Graduate School of Medicine, Kyoto University, Kyoto, Japan; 2 Center for Innovation in Immunoregulative Technology and Therapeutics, Graduate School of Medicine, Kyoto University, Kyoto, Japan; 3 Research Portfolio & Science, Astellas Pharma Inc., Tokyo, Japan; 4 Department of Nephrology, Graduate School of Medicine, Kyoto University, Kyoto, Japan; 5 Department of Rheumatology and Clinical Immunology, Wakayama Medical University, Wakayama, Japan; Nippon Medical School, JAPAN

## Abstract

We assessed the utility of two forms of osteopontin (OPN), OPN full and its cleaved form (OPN N-half), in plasma and urine as markers of disease activity in lupus nephritis (LN). Samples were collected from patients with systemic lupus erythematosus (SLE) (LN: N = 29, non-LN: N = 27), IgA nephropathy (IgAN) (N = 14), minimal change nephrotic syndrome (MCNS) (N = 5), diabetic nephropathy (DN) (N = 14) and healthy volunteers (HC) (N = 17). While there was no significant difference in urine OPN full concentration between groups, urine OPN N-half concentration was significantly higher in patients with LN than HC (*p* < 0.05). Moreover, urine OPN N-half was higher in LN patients with overt proteinuria (urine protein/creatinine ratio: P/C > 0.5) than LN patients with minimal proteinuria (P/C < 0.5, *p* < 0.0001), and also higher than in DN patients with overt proteinuria (P/C > 0.5, *p* < 0.01). Urine thrombin activity correlated with urine OPN N-half concentration (*p* < 0.0001), but not with urine OPN full concentration. These results suggest that urine OPN N-half concentration reflects renal inflammation. Thus, urine OPN N-half may be a novel disease activity marker for LN.

## Introduction

Systemic lupus erythematosus (SLE) is an autoimmune disease characterized by autoreactive B and T cells and autoantibodies, including anti-nuclear and anti-DNA antibodies. Lupus nephritis (LN) is one of multiple organ manifestations of SLE, and is an important prognostic factor [1]; therefore, new, improved therapeutic approaches and biomarkers are needed. Urine protein and serum creatinine (Cre) are traditionally used as markers of LN. As SLE patients may have several complicating nephropathies in the long course of treatment, including LN, drug-induced nephropathy, and diabetic nephropathy, new markers that can discriminate between these complicated renal lesions are needed.

Osteopontin (OPN) is a secretory glycoprotein, whose molecular mass ranges from 44–66 kDa due to variation in glycosylation [2]. OPN is expressed by osteoblasts, macrophages, activated T cells, and distal tubular epithelial cells. Notably, OPN produced by osteoblasts can activate bone resorption by osteoclasts [3, 4].

OPN is considered important in both normal and dysregulated immune responses as it promotes infiltration of macrophages and T cells to inflammatory sites [5, 6]. Full-length OPN (OPN full) is cleaved by proteases including thrombin and matrix metalloproteinase (MMP)-3, and the N-terminal fragment of the cleaved OPN (OPN N-half) accelerates immune cell infiltration [6]. Tahir et al. reported that senescent T cells are increased in BWF1 mice, a known lupus-prone animal model. They also demonstrated that OPN secreted by senescent T cells inhibits the apoptosis of B cells and assists in the production of autoantibodies and formation of germinal centers [7].

Moreover, OPN may play an important role in the pathophysiology of SLE. The expression level of OPN in distal tubular epithelium is correlated with both serum creatinine and the number of monocytes infiltrating into renal interstitial tissues of patients with various forms of glomerulonephritis, including LN [8]. Several articles have reported that the concentration of urine and serum OPN is increased in patients with SLE and correlates with disease activity. Wong and Liu reported higher OPN levels in plasma and urine of SLE patients compared with healthy controls (HC) [9, 10], although they did not distinguish between OPN full and OPN N-half.

While the above reports imply an association of OPN with the pathophysiology of SLE, especially with lupus nephritis, the association of OPN full and OPN N-half with lupus pathophysiology has not been described. In this study, we measured the concentration of OPN in plasma and urine of HC, non-LN SLE, LN, and other renal diseases with two ELISA systems identifying OPN full and OPN N-half, respectively. We assessed the roles of the two forms of OPN in the pathophysiology of LN, and the utility of their plasma and urine concentration as markers of disease activity in LN.

## Materials and Methods

### Patients and healthy controls

We collected plasma and urine from 56 randomly selected patients with SLE (29 patients with LN, 27 patients without LN: non-LN), 14 patients with diabetic nephropathy (DN), 5 with minimal change nephrotic syndrome (MCNS) and 14 patients with IgA nephropathy (IgAN), who visited the Department of Rheumatology and Clinical Immunology or the Department of Nephrology, Kyoto University Hospital. Patients’ clinical information was obtained from medical records. The diagnosis of SLE was based on the 1997 revised classification criteria of the American College of Rheumatology (ACR) [11, 12]. The diagnosis of DN was based on a history of diabetes mellitus, proteinuria, and histopathology. The diagnosis of MCNS was based on nephrosis and minimal histopathological findings. The diagnosis of IgAN was based on histopathology findings demonstrating glomerulonephritis with predominant IgA deposits in the mesangium without systemic disease [13]. We also collected plasma and urine from 17 healthy volunteers (HC) who were matched for the mean age and sex ratio of SLE patients. The study protocol was accepted by the Ethics Committee, Kyoto University Graduate School and Faculty of Medicine (G489; Jul 4, 2012). We obtained written informed consent from all patients and volunteers before sampling.

### Plasma and urine samples

Plasma and urine samples were collected from each patient and HC and placed on ice. Halt™ Protease Inhibitor Single-Use Cocktail (Thermo Fisher Scientific, Inc., Waltham, MA, USA) was added to urine in which OPN full and OPN N-half were to be measured. All urine and plasma samples were stored at −80°C and thawed just before the performance of assays.

### Assays

The concentration of OPN full, OPN N-half, and urine creatinine was measured by Human Osteopontin Assay Kit (IBL Co. Ltd., Gunma, Japan) using Recombinant Human Osteopontin Protein (R & D Systems Inc., Minneapolis, MN, USA) as a standard, Human Osteopontin N-half Assay Kit (IBL), and Urinary Creatinine ELISA Kit (Trans Genic Inc. Ltd., Fukuoka, Japan), respectively. The Human Osteopontin N-Half Assay Kit specifically measures the levels of the N-terminal OPN fragment cleaved by thrombin. In contrast, OPN molecules without thrombin cleavage are minimally detected by the kit, according to the manufacturer’s datasheet. The Human Osteopontin (full) Assay Kit recognizes both the N-terminal (IPVKQADSGSSEEKQ) and C-terminal (KSKKFRRPDIQYPDATDE) of full-length osteopontin, indicating that OPN N-half is not recognized by this kit.

Urine protein was measured by Bradford assay. The activity of urine thrombin and MMP-3 was measured by SensoLyte^®^ 520 Thrombin Activity Fluorimetric Assay Kit (Anaspec, Inc., Fremont, CA, USA) and MMP-3 Activity Assay Kit (COSMO BIO Co. Ltd., Tokyo, Japan), respectively. The concentration of renal damage markers, namely, albumin (ALB), β2-microglobulin (B2M), cystatin C, calbindin, glutathione S-transferase pi (GST-π), kidney injury molecule 1 (KIM-1), neutrophil gelatinase-associated lipocalin (NGAL), clusterin, trefoil factor 3 (TFF3), interleukin 18 (IL-18), and monocyte chemoattractant protein 1 (MCP-1), in urine were measured by Bio-Plex Pro™ RBM Human Kidney Toxicity Assays (Bio-Rad Laboratories, Inc., Hercules, CA, USA).

### Statistical analysis

Statistical analysis was performed using GraphPad Prism 5 software (GraphPad Software Inc., La Jolla, CA, USA). We used ANOVA corrected by the Bonferroni method when the variance of each group was assumed to be similar by Bartlett analysis, and we used Dunn’s multiple comparisons test when the variance of each group was not assumed to be similar by Bartlett analysis. Pearson’s correlation test was used for correlation analysis. P-values of *p* < 0.05 were deemed to indicate statistically significant differences.

## Results

### Profiles of patients

We enrolled patients with SLE (29 with LN: LN, 27 without LN: non-LN), DN (14), MCNS (5) and IgAN (14) at random (Table 1). The patients with SLE showed a variety of disease activity indices (SLEDAI), histopathological classes of International Society of Nephrology/Renal Pathology Society (ISN/RPS) and histopathological activity/chronicity indices of National Institutes of Health (NIH). Most of the SLE patients were treated with glucocorticoids and various immunosuppressants.

**Table 1:** Profiles of patients and healthy controls.

	HC	Non-LN	LN	DN	MCNS	IgAN
Diagnosis	Healthy volunteers	SLE without LN	SLE with LN	Diabetic nephropathy	Minimal change nephrotic syndrome	IgA nephropathy
Number	17	27	29	14	5	14
Sex ratio (male/female)	0:17	1:26	1:28	10:4	2:3	6:8
Age (range)	37 ± 2 (23–50)	44 ± 3 (23–78)	38 ± 3 (19–78)	65 ± 4 (31–83)	47 ± 10 (18–66)	49 ± 4 (26–76)
SLEDAI (range)	-	3.3 ± 0.9 (0–21)	4.0 ± 0.9 (0–17)	-	-	-
Serum Cre (mg/dl)	-	0.63 ± 0.03	0.69 ± 0.04	2.45 ± 0.48	0.76 ± 0.10	1.10 ± 0.13
Urine Protein (g/gCre)	0.09 ± 0.02	0.15 ± 0.03	0.78 ± 0.23	1.82 ± 0.44	1.59 ± 0.57	1.00 ± 0.30
Histopathology	-	-	• ISN/RPS class: II (4), III (2), IV (7), V (6), Mixed (2), Unknown (2), Not biopsied (6)	• Clinically and/or histopathologically diagnosed	• Minimal histopathological findings	• Predominant IgA deposits in mesangium
			• NIH-AI: 6.0 ± 1.8 (10)			
			• NIH-CI: 1.4 ± 1.0 (10)			
Treatments	-	• GCs: 25 (PSL 10.0 ± 10.4 mg/day)	• GCs: 28 (PSL 9.1 ± 5.0 mg/day)	• GCs: 0	• GCs: 2 (PSL 6.3 ±1.3 mg/day)	• GCs: 2 (PSL 7.5 ± 2.5 mg/day)
		• TAC: 3	• TAC: 10		• CSA: 1	
		• CSA: 1	• CSA: 2		• MZR: 1	
		• MZR: 1	• MZR: 5			
		• AZA: 3	• AZA: 1			
		• IVCY: 1	• IVCY: 1			
		• POCY: 1				
		• MTX: 1				

The ISN/RPS classes of 23 patients with LN who were biopsied in either our hospital or other hospitals are shown. The NIH-AI and CI of 10 patients whose renal specimens were available for re-evaluation are indicated. The dose of GCs was calculated as the dose of PSL. Values are expressed as the mean ± SE. Abbreviations: AI, activity index; AZA, azathioprine; CI, chronicity index; Cre, creatinine; CSA, cyclosporine A; GCs, glucocorticoids; ISN/RPS, The International Society of Nephrology/Renal Pathology Society; IVCY, intravenous cyclophosphamide; MTX, methotrexate; MZR, mizoribine; NIH, National Institutes of Health; POCY, oral cyclophosphamide; PSL, prednisolone; SLEDAI, systemic lupus erythematosus disease activity index; TAC, tacrolimus.

While 23 of 29 LN patients (79.3%) were biopsy-proven, the other 6 patients (20.7%) were not biopsied. The details of the 6 patients are shown in S1 Table. All 6 patients fulfilled the criteria for SLE. Although we could not perform renal biopsy for compelling reasons, including thrombocytopenia, use of anticoagulants, psychosis and pregnancy, patients were diagnosed as having LN based on the presence of proteinuria, urinary casts, microhematuria, hypoalbuminemia, and coexisting elevation of anti-DNA autoantibodies.

### Urine concentration of OPN N-half and OPN full

**Fig 1:**
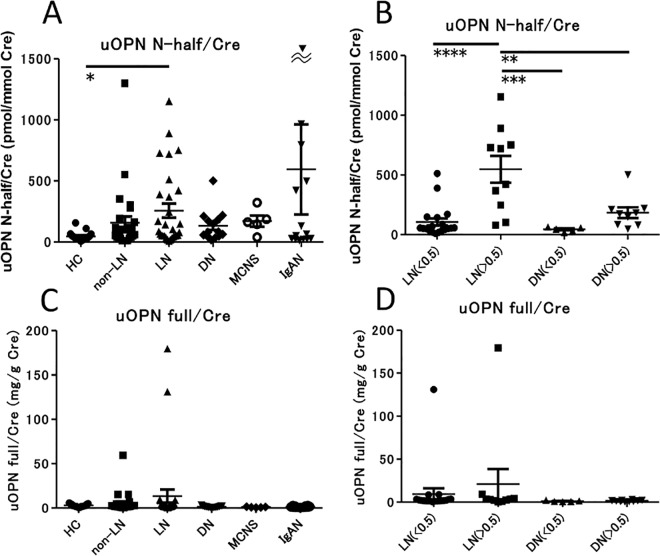
Comparison of the concentration of urine OPN full and N-half in SLE and several renal diseases. Concentration of urine OPN N-half (A) and urine OPN full (C) in healthy controls (N = 17) and patients with SLE without nephropathy (N = 27), LN (N = 29), DN (N = 14), MCNS (N = 5) and IgAN (N = 14). Values were corrected by calculating the ratio to urine creatinine (Cre) concentration. * *p* < 0.05 by Dunn’s test. Concentration of urine OPN N-half (B) and urine OPN full (D) in patients with LN and DN, separated into subgroups with minimal (P/C ratio < 0.5) or overt (P/C ratio > 0.5) proteinuria. Values are corrected by urine Cre level. ** *p* < 0.01, *** *p* < 0.001, **** *p* < 0.0001 by ANOVA corrected by the Bonferroni method.

We initially measured the concentration of OPN N-half and OPN full in urine (Fig 1). Urine OPN N-half concentration was significantly increased in patients with LN compared with HC (*p* < 0.05) (Fig 1A). We divided the LN and DN groups into two subgroups of patients with overt proteinuria (urine protein/creatinine ratio (P/C) > 0.5) and patients with minimal proteinuria (urine P/C < 0.5). Urine OPN N-half was higher in LN patients with overt proteinuria than LN patients with minimal proteinuria (*p* < 0.0001). Interestingly, urine OPN N-half was higher in LN patients with overt proteinuria than in DN patients with overt proteinuria (*p* < 0.01) (Fig 1B). In contrast, there were no significant differences in urine OPN full concentration between the groups (Fig 1C). There were also no significant differences in urine OPN full concentration between the LN and DN groups as subdivided by P/C (Fig 1D). Urine OPN N-half was also higher in IgAN with overt proteinuria than IgAN with minimal proteinuria (S1A Fig), similar to LN.

**Fig 2:**
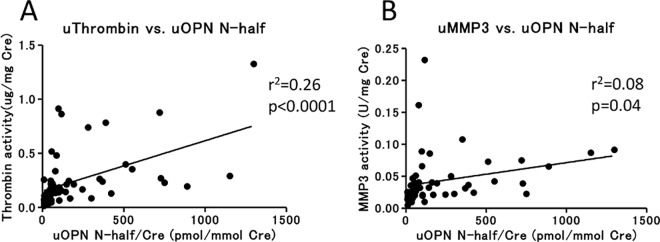
Correlation of OPN N-half level with thrombin or MMP-3 activity in urine. Correlation of urine OPN N-half level with urine thrombin activity (A) or urine MMP-3 activity (B) in SLE patients (N = 56). Values were corrected by calculating the ratio to urine Cre concentration. r^2^: coefficient of determination of a linear regression analysis.

To assess whether OPN N-half can be used as a marker to identify the site of damage to the nephron, we examined the correlation of urine OPN N-half with markers of proximal tubule damage (ALB, B2M, cystatin C, KIM-1, and NGAL), distal tubule damage (calbindin and GST-π), and general inflammation (IL-18 and MCP-1) in the SLE patients. We found that urine OPN N-half was correlated with IL-18, as well as ALB (Table 2, S2 Fig). Next, we measured urine thrombin and MMP-3 activity, which cleave OPN full to yield OPN N-half. Urine OPN N-half correlated with urine thrombin activity, but not with urine MMP-3 activity (Fig 2).

**Table 2:** Correlation of urine OPN N-half with renal damage markers.

	Urine OPN full	Plasma OPN full	Total Protein	Albumin	IL-18	MCP-1	B2M	Cystatin C	Calbindin	GST-π	KIM-1	NGAL	Clusterin	TFF3
R	0.08	0.19	0.44	0.67	0.64	0.39	0.25	0.27	0.27	0.42	0.34	0.33	0.38	0.19
p-value	0.56	0.17	0.0006	<0.0001	<0.0001	0.003	0.06	0.04	0.04	0.001	0.01	0.013	0.004	0.15

R: Pearson product-moment correlation coefficient. When R was more than 0.45, the values were considered to be correlated. B2M, β2-microglobulin; GST-π, glutathione S-transferase pi; KIM-1, kidney injury molecule-1; MCP-1, monocyte chemoattractant protein 1; NGAL, neutrophil gelatinase-associated lipocalin; TFF3, trefoil factor 3.

**Fig 3:**
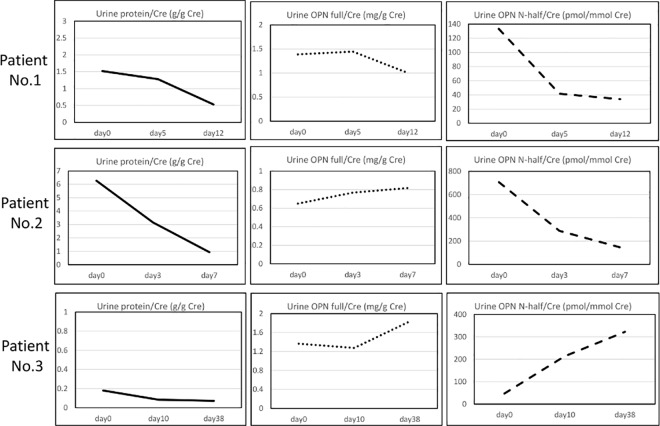
Time course of urine protein, OPN full and OPN N-half concentration in three patients with LN treated with immunosuppressive therapy. There were three patients with LN from whom we could obtain serial samples before and after treatment. On Day 0, prednisolone (1 mg/kg/day) was started.

We next assessed the changes in urine OPN N-half concentration over the course of treatment of three patients with LN (Fig 3), in whom we were able to obtain serial samples, before and after treatment. In two cases with overt proteinuria, urine OPN N-half was decreased less than a week after increasing the dose of glucocorticoids. In the other patient, who did not show proteinuria, urine OPN N-half concentration was elevated after treatment.

**Fig 4:**
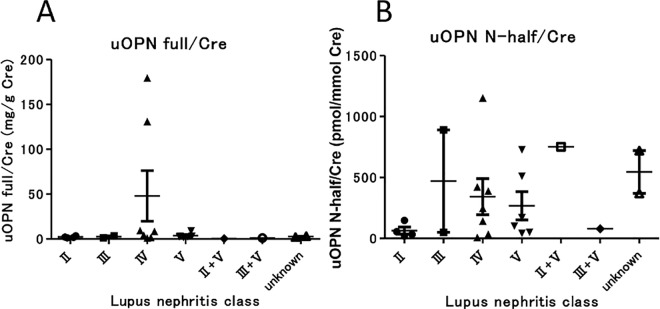
Association of urine OPN full and urine OPN N-half with ISN/RPS 2003 histopathological classification of lupus nephritis. Urine OPN full tended to be higher in class IV LN than in other classes (A), and urine OPN N-half level was lower in class II LN than in other classes (B), although there were no statistically significant differences among classes by Dunn’s test.

We assessed the correlation between urine OPN N-half and histopathological grading (ISN/RPS classification of LN) of biopsied kidneys (Fig 4). We found that urine OPN N-half tended to be low in class II LN. In contrast, urine OPN N-half showed no differences among class III/IV/V of lupus nephritis. We also analyzed the correlation between NIH-Activity/Chronicity Indices and urine OPN N-half (S3 Fig); however, we found no significant correlations between them.

SLE patients may have DN as a complication during the course of treatment. We measured urine OPN N-half concentration in a female SLE patient complicated with diabetes. Disease activity of SLE was considered low according to clinical manifestations and serum C3 (121.9 mg/dl), C4 (38.5 mg/dl), CH50 (53 U/ml) and anti-DNA antibody (2.9 IU/ml) levels, although she showed overt proteinuria (P/C = 3.4). The patient had been treated with insulin and nateglinide, which were stopped 6 years prior to the collection of plasma for this study. At the time of collection, HbA1c was 6.0%. Urine OPN N-half/Cre was 60.4 pmol/mmol Cre, which was as low as healthy controls, corresponding with her low SLE activity.

### Plasma concentration of OPN full and OPN N-half

**Fig 5:**
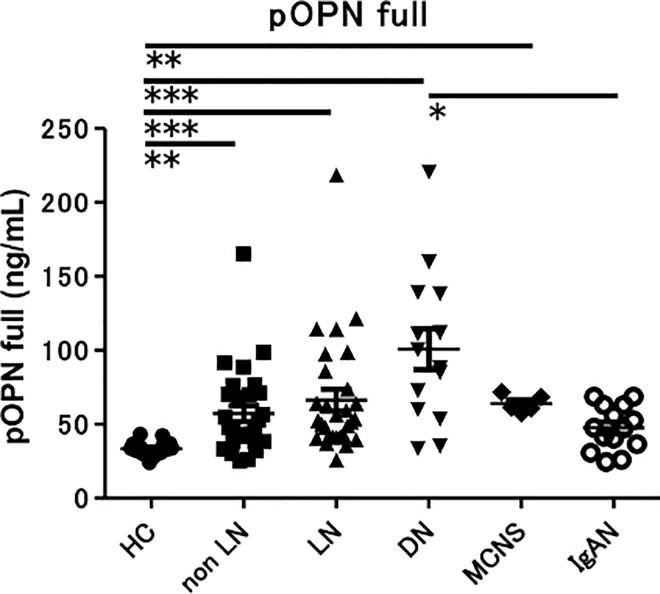
Concentration of plasma OPN full in SLE and various renal diseases. Concentration of plasma OPN full in healthy controls (N = 17) and patients with SLE without nephropathy (N = 27), lupus nephritis (N = 29), DN (N = 14), MCNS (N = 5) and IgAN (N = 14). ** *p* < 0.01, *** *p* < 0.001 by Dunn’s test.

Plasma OPN full concentration was significantly higher in LN, non-LN, DN and MCNS than in HC (*p* < 0.01, *p* < 0.001) (Fig 5). Unexpectedly, plasma OPN N-half concentration in all groups was below the detection sensitivity (data not shown). We also divided the LN and DN groups into two subgroups of patients with urine P/C > 0.5 and patients with urine P/C < 0.5. There were no significant differences in plasma OPN full concentration between the subgroups (S4 Fig).

### Correlation between OPN and systemic disease activity markers

We assessed the correlation of plasma and urine OPN full and N-half concentration with systemic disease activity markers in the SLE patients. Intriguingly, the concentration of plasma and urine OPN full were significantly correlated with anti-dsDNA antibody titer; however, the concentration of urine OPN N-half was not (S5A Fig). Plasma and urine OPN full and N-half concentration were not correlated with other systemic disease activity markers including SLEDAI (SLE disease activity index) (S5B Fig), C3, C4, and CH50 (data not shown). They were also not correlated with the eGFR (estimated glomerular filtration rate) (S5C Fig) or dosage of glucocorticoids (data not shown).

## Discussion

There have been several studies reporting that OPN is increased in the plasma and urine of patients with SLE, and that plasma and urine OPN concentration correlates with the disease activity of SLE [9, 10]. In those studies, however, they did not distinguish between OPN full and OPN N-half. Therefore, in this study, we used two ELISA systems identifying OPN full and OPN N-half, respectively. To the best of our knowledge, this is the first report that assessed the roles of OPN full versus OPN N-half in SLE. We found that urine OPN N-half was higher in active LN patients (P/C > 0.5) than in stable LN (P/C < 0.5) (Fig 1B), and that there was a significant correlation between urine protein and urine OPN N-half concentration (Table 2, S2 Fig).

Urine OPN N-half in the LN patients with overt proteinuria was higher than in the DN patients with overt proteinuria (Fig 1B). Generally, inflammatory cell infiltration is observed in the renal histopathology of LN patients, while it is not observed in DN patients. Moreover, when we measured renal damage markers in the urine of patients with LN to assess whether urine OPN N-half could be a marker that can identify the site of nephron damage, we observed urine OPN N-half was correlated with urine IL-18 (Table 2, S2 Fig), suggesting that urine OPN N-half was correlated with overall renal inflammation, and not with damage to a specific nephron section, and so probably reflected the activity of LN.

We therefore hypothesized that urine OPN N-half is produced by the cleavage of OPN full due to renal inflammation. It has been reported previously that both thrombin and MMP-3 are activated in the pathophysiology of glomerulonephritis [14–17]. We measured protease activity in urine and found that urine OPN N-half concentration correlated with urine thrombin activity (Fig 2A). In contrast, urine OPN N-half was not correlated with urine MMP-3 activity (Fig 2B). Notably, the enzyme cleavage site of OPN full is different between thrombin and MMP-3 [18, 19], and the ELISA antibodies that we used to measure OPN N-half concentration in this study recognizes an amino acid sequence (SVVYGLR^162-168^) located at the cutting edge of OPN N-half as cleaved by thrombin [20]. MMP-3 cleaves three other points of OPN, one is between G^166^ and L^167^ and the others are at the C-terminal side [18]. The OPN fragments cleaved by MMP-3 cannot be recognized by this ELISA system because the SVVYGLR sequence is lost or hidden. Therefore, the urine OPN N-half concentration that we measured was correlated only with urine thrombin activity, but not with urine MMP-3 activity.

There is a possibility that OPN N-half leaks through the glomerular basement membrane as a urinary protein. In other words, urine N-half concentration was merely proportionally correlated with urine protein concentration. Therefore, we analyzed the urine OPN N-half concentration normalized by urine protein concentration (S6 Fig). Notably, there was a significant difference in urine OPN N-half/urine protein ratio between HC and SLE patients, although we did not find differences in urine OPN N-half/urine protein ratio between SLE patients with and without LN (S6B Fig). However, we consider that urine OPN N-half concentration is not merely proportionally correlated with urine protein concentration, because i) OPN N-half could not be detected in plasma and ii) we demonstrated that urine OPN N-half concentration was high in LN patients with massive proteinuria, while it was low in DN patients with massive proteinuria (Fig 1B). Because DN is a renal disease with less inflammation compared with LN, the cleavage of OPN full by thrombin (Fig 2A) might be due to renal inflammation in LN. We consider that both urine protein and urine OPN N-half correlate with disease activity in LN (S7 Fig).

There were no significant differences in urine OPN full concentration between the groups (Fig 1C). In contrast to our results, urine OPN full was increased in SLE and correlated with disease activity in previous studies [10]. This may be because the ELISA system used in those studies might also be able to detect OPN N-half. From our results, urine OPN N-half, but not urine OPN full, is a marker that correlates with disease activity in LN.

In this study, plasma OPN full in SLE was higher than in HC (Fig 5). Rullo et al. reported that plasma OPN was increased and associated with organ damage and disease activity in SLE [21]. There have been several studies that reported plasma OPN full is increased by inflammation such as injury and infection [2]. OPN is secreted by proinflammatory cells including activated T cells [22] and macrophages [23] and Denhardt et al. reported that gene expression levels of OPN are upregulated by inflammatory cytokines such as IL-1β, IL-6, interferon (IFN)-α and IFN-γ [24]. Plasma cytokine concentrations are elevated in SLE patients, and thus OPN might be secreted from activated T cells and macrophages infiltrating into the affected organs.

In our data, urine OPN N-half was higher in IgAN with overt proteinuria than IgAN with minimal proteinuria (S1B Fig), similar to LN. Gang et al. reported that thrombin-cleaved OPN fragments in urine are correlated with urine protein level in IgAN [16], and presumably, OPN N-half corresponds to the thrombin-cleaved OPN fragment in their report. We therefore hypothesize that urine OPN N-half level is increased as a result of renal inflammation in both LN and IgAN. It is also consistent with there being no significant differences in urine OPN N-half concentrations between LN class III/IV and IgAN (S1B Fig). Thus, increased urine OPN N-half is considered not specific to LN, but still useful to distinguish inflammatory renal disease from non-inflammatory renal disease.

We assessed whether urine OPN N-half level can predict the histopathological class of LN as defined by the ISN/RPS classification [25, 26]. Urine OPN N-half tended to be lower in LN class II than in other classes (Fig 4B). This might be because urine OPN N-half concentration reflects inflammation of the kidney in LN and inflammation is milder in the histopathology of LN class II compared with other classes.

In our study, plasma OPN full levels in patients with SLE, DN, and MCNS were increased (Fig 5). Yamaguchi et al. reported that serum OPN is significantly increased in diabetic patients with elevated serum creatinine levels of more than 2 mg/dl compared with HC [27]. In our data, serum creatinine level was 2.45 ± 0.48 mg/dl (mean ± SE) in patients with DN. The increased plasma OPN concentration in diabetic patients was considered to be caused by decreased renal excretion. It has also been reported that T cells and IL-2 are associated with the pathophysiology of MCNS [28, 29], and the proinflammatory conditions may be linked to increased plasma OPN level in patients with MCNS.

Urine OPN N-half was not correlated with systemic disease activity markers, including anti-dsDNA antibody (S5A Fig) and SLEDAI (S5B Fig). However, this is not unexpected, given that the SLEDAI score can be high because of the involvement of organs other than the kidney.

We have shown cross-sectional data in regard to urine OPN concentration (e.g. Fig 1). Next, we examined urine OPN N-half concentration in the clinical course of LN (Fig 3). Urine OPN N-half concentration was decreased after the dose of glucocorticoids was increased in 2 LN patients with overt proteinuria. That might be because the renal thrombin activity was decreased by glucocorticoid therapy. In the other patient, urine OPN N-half concentration was unexpectedly elevated after treatment, however, that might be because the activity of renal inflammation had been low before the treatment.

SLE patients occasionally have DN as a complication during treatment. As we described in the Results, we treated a female SLE patient complicated with diabetes. As her SLE disease activity and urine OPN N-half/Cre ratio were low, the proteinuria demonstrated is due to DN, not LN. This indicates the measurement of urine OPN N-half might be useful to assess the cause of proteinuria.

## Conclusions

We evaluated the use of two forms of OPN in the diagnosis of patients with LN. Urine OPN N-half concentration was higher in LN patients than in healthy controls. Moreover, LN patients with overt proteinuria had higher concentrations of urine OPN N-half than those without, suggesting that urine OPN N-half reflects inflammation of the kidney. Moreover, urine OPN N-half level was lower in patients with DN showing overt proteinuria than in patients with LN showing overt proteinuria. Therefore, although increased urine OPN N-half is not specific to LN, it is useful to distinguish inflammatory renal disease from non-inflammatory renal disease. These results suggest that urine OPN N-half may be a novel marker of disease activity in patients with LN.

## Supporting Information

S1 Fig Comparison of urine OPN N-half levels between LN and IgAN. Concentration of urine OPN N-half corrected by urine Cre level in patients with LN and IgA nephropathy (IgAN) with minimal (P/C ratio < 0.5) or overt (P/C ratio > 0.5) proteinuria (A). Comparison of urine OPN N-half levels between LN class III/IV and IgAN (B). * *p* < 0.05 by ANOVA corrected by the Bonferroni method.
(TIF)

S2 Fig Correlation of urine OPN N-half concentration with renal damage markers. Values are corrected by calculating ratio to urine Cre concentration. r^2^: coefficient of determination of a linear regression analysis. ALB: albumin, B2M: β2-microglobulin, Cyst C: cystatin C, CLBN: calbindin, GST-π: glutathione S-transferase pi, KIM-1: kidney injury molecule-1, NGAL: neutrophil gelatinase-associated lipocalin, CLSN: clusterin, TFF3: trefoil factor 3, MCP-1: monocyte chemoattractant protein 1.
(TIF)

S3 Fig Correlation between NIH-Activity/Chronicity Indices and urine OPN N-half. We analyzed the correlations in 10 cases whose renal specimens were available for re-evaluation. The 4 cases in whom the timing of urine collection and renal biopsy was close are indicated by filled circles, while the other 6 cases are indicated by open circles. AI: activity index, CI: chronicity index.
(TIF)

S4 Fig Comparison of plasma OPN full between LN and DN patients. Concentration of plasma OPN full in patients with LN and DN with minimal (P/C ratio < 0.5) or overt (P/C ratio > 0.5) proteinuria. There were no significant differences among groups by ANOVA corrected by the Bonferroni method.
(TIF)

S5 Fig Correlation of OPN full/N-half with clinical data. Correlation of titer of anti-dsDNA antibody (A), SLE disease activity index (SLEDAI) (B), and estimated glomerular filtration ratio (eGFR) (C) with plasma OPN full, urine OPN full, or urine OPN N-half levels in SLE patients (N = 56). Urine OPN full and N-half levels are corrected by calculating ratio to urine Cre concentration. r^2^: coefficient of determination of a linear regression analysis.
(TIF)

S6 Fig Comparison of urine OPN full/N-half corrected by urine protein. Concentration of urine OPN full (A) and urine OPN N-half (B) corrected by urine protein level in healthy controls (HC), SLE patients without LN (Non-LN), and those with LN. There was a significant difference in urine OPN N-half/urine protein ratio between HC and SLE patients (* *p* < 0.05 by Dunn’s test), however we did not find differences between SLE patients with and without LN.
(TIF)

S7 Fig Mechanisms of correlation between proteinuria and urine OPN N-half in active LN. In active glomerulonephritis, the leakage of protein into urine is increased because of the impairment of glomerular barrier function as a result of inflammation around the GBM. Activation of thrombin is therefore increased locally at the same time. Activated thrombin can then cleave OPN in the urine. GBM: glomerular basement membrane.
(TIF)

S1 Table Six unbiopsied cases diagnosed as having LN. All 6 patients fulfilled the criteria for SLE. Although we could not perform renal biopsy for compelling reasons, they were clinically diagnosed as having LN.
(TIF)
