# Supplementary figures and images for: Cleaved Form of Osteopontin in Urine as a Clinical Marker of Lupus Nephritis
Source: PLoS One. 2016 Dec 19;11(12):e0167141. doi: 10.1371/journal.pone.0167141 (PMC5167225; doi:10.1371/journal.pone.0167141)

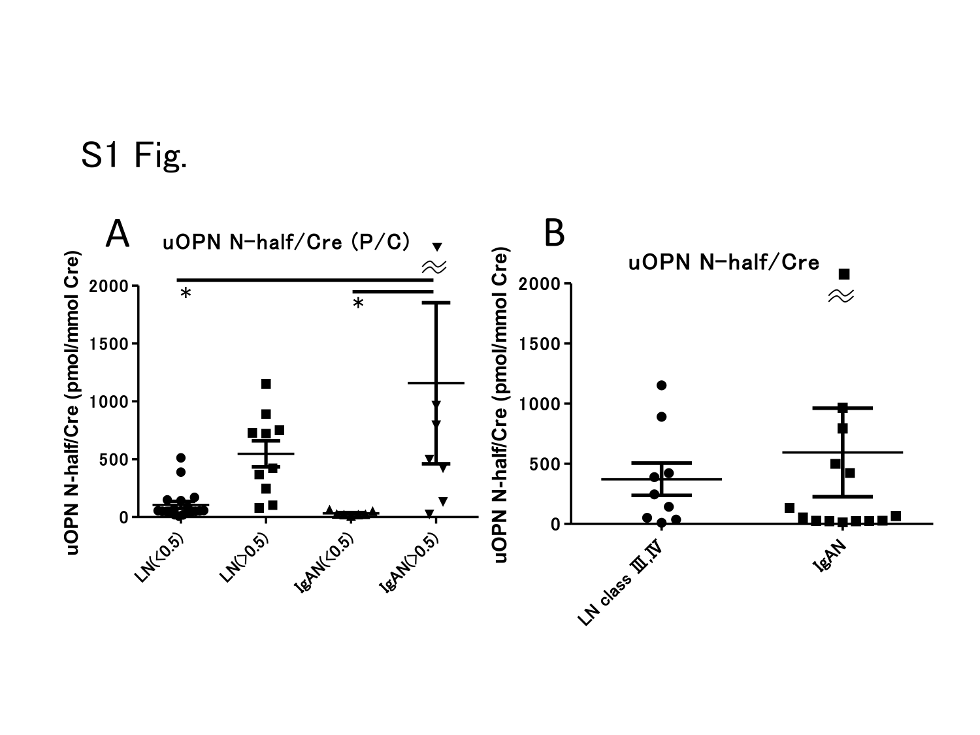

Supplement: S1 Fig — Concentration of urine OPN N-half corrected by urine Cre level in patients with LN and IgA nephropathy (IgAN) with minimal (P/C ratio < 0.5) or overt (P/C ratio > 0.5) proteinuria (A). Comparison of urine OPN N-half levels between LN class III/IV and IgAN (B). * p < 0.05 by ANOVA corrected by the Bonferroni method. (TIF) [file pone.0167141.s001.tif]

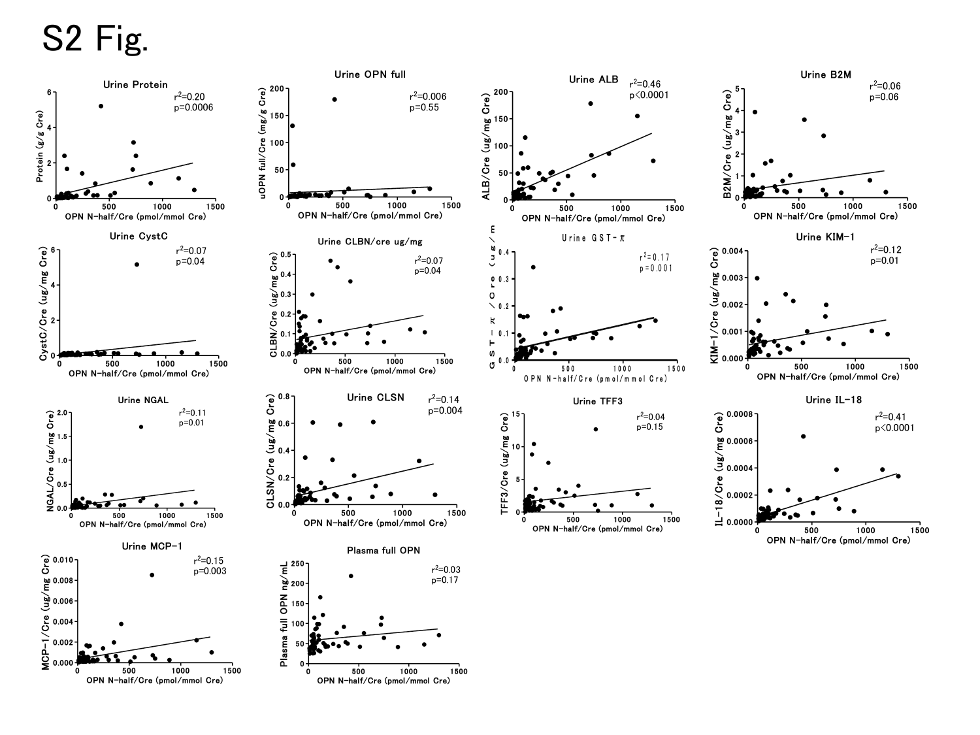

Supplement: S2 Fig — Values are corrected by calculating ratio to urine Cre concentration. r2: coefficient of determination of a linear regression analysis. ALB: albumin, B2M: β2-microglobulin, Cyst C: cystatin C, CLBN: calbindin, GST-π: glutathione S-transferase pi, KIM-1: kidney injury molecule-1, NGAL: neutrophil gelatinase-associated lipocalin, CLSN: clusterin, TFF3: trefoil factor 3, MCP-1: monocyte chemoattractant protein 1. (TIF) [file pone.0167141.s002.tif]

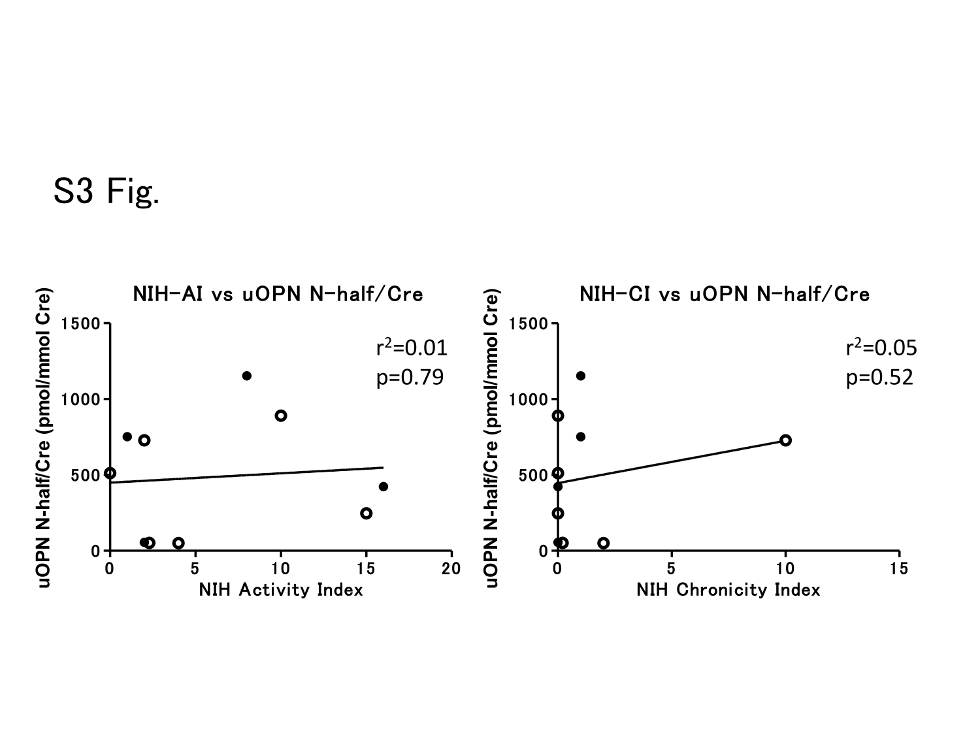

Supplement: S3 Fig — We analyzed the correlations in 10 cases whose renal specimens were available for re-evaluation. The 4 cases in whom the timing of urine collection and renal biopsy was close are indicated by filled circles, while the other 6 cases are indicated by open circles. AI: activity index, CI: chronicity index. (TIF) [file pone.0167141.s003.tif]

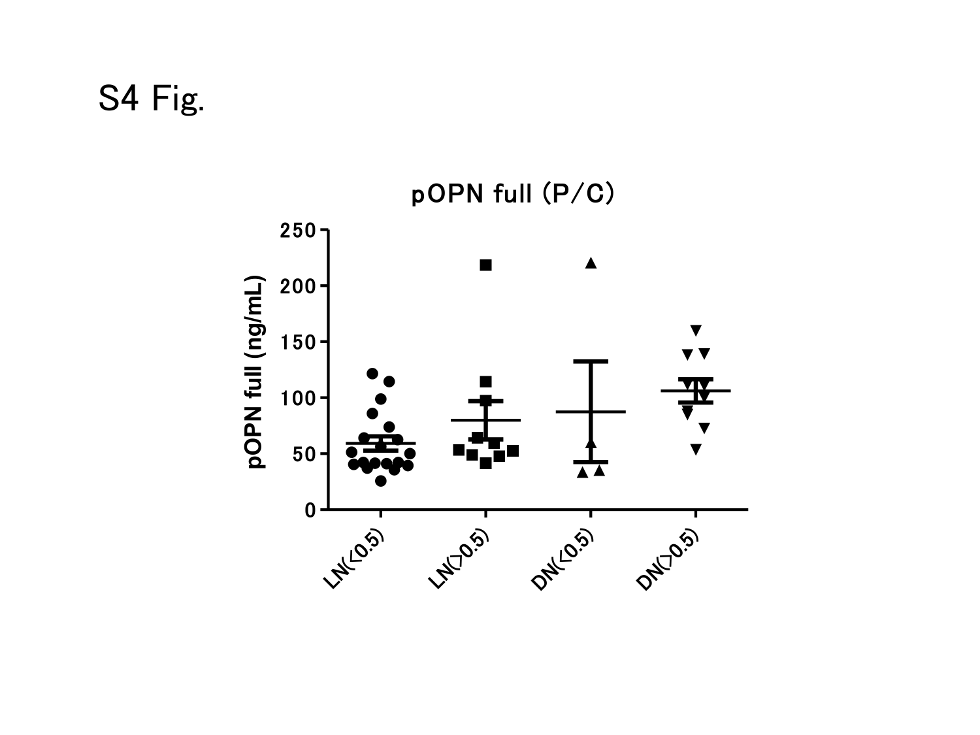

Supplement: S4 Fig — Concentration of plasma OPN full in patients with LN and DN with minimal (P/C ratio < 0.5) or overt (P/C ratio > 0.5) proteinuria. There were no significant differences among groups by ANOVA corrected by the Bonferroni method. (TIF) [file pone.0167141.s004.tif]

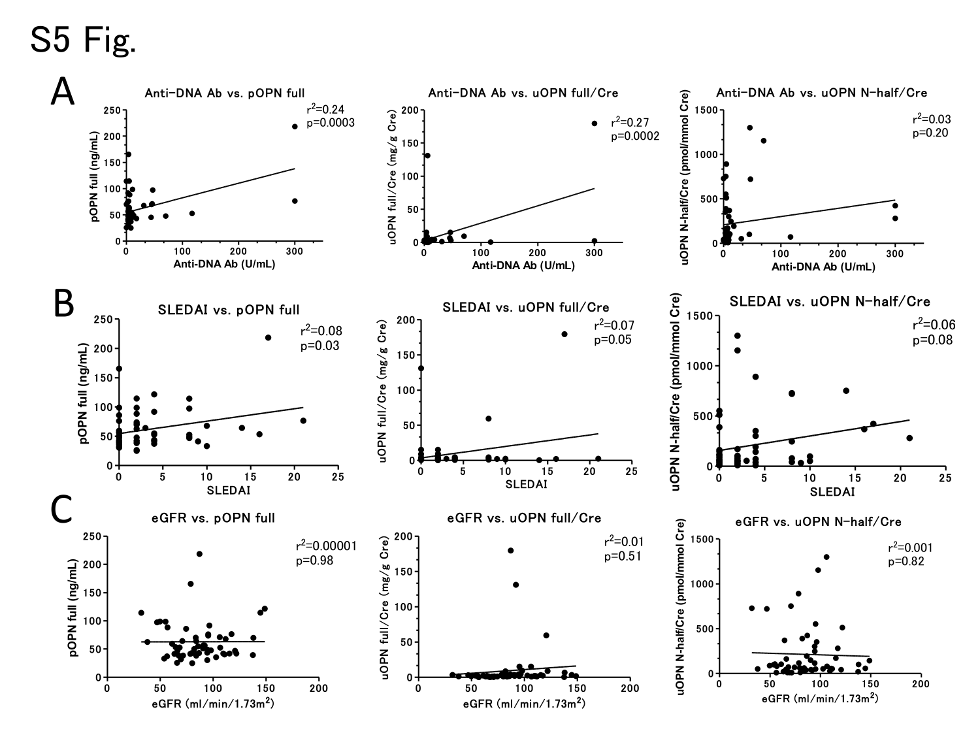

Supplement: S5 Fig — Correlation of titer of anti-dsDNA antibody (A), SLE disease activity index (SLEDAI) (B), and estimated glomerular filtration ratio (eGFR) (C) with plasma OPN full, urine OPN full, or urine OPN N-half levels in SLE patients (N = 56). Urine OPN full and N-half levels are corrected by calculating ratio to urine Cre concentration. r2: coefficient of determination of a linear regression analysis. (TIF) [file pone.0167141.s005.tif]

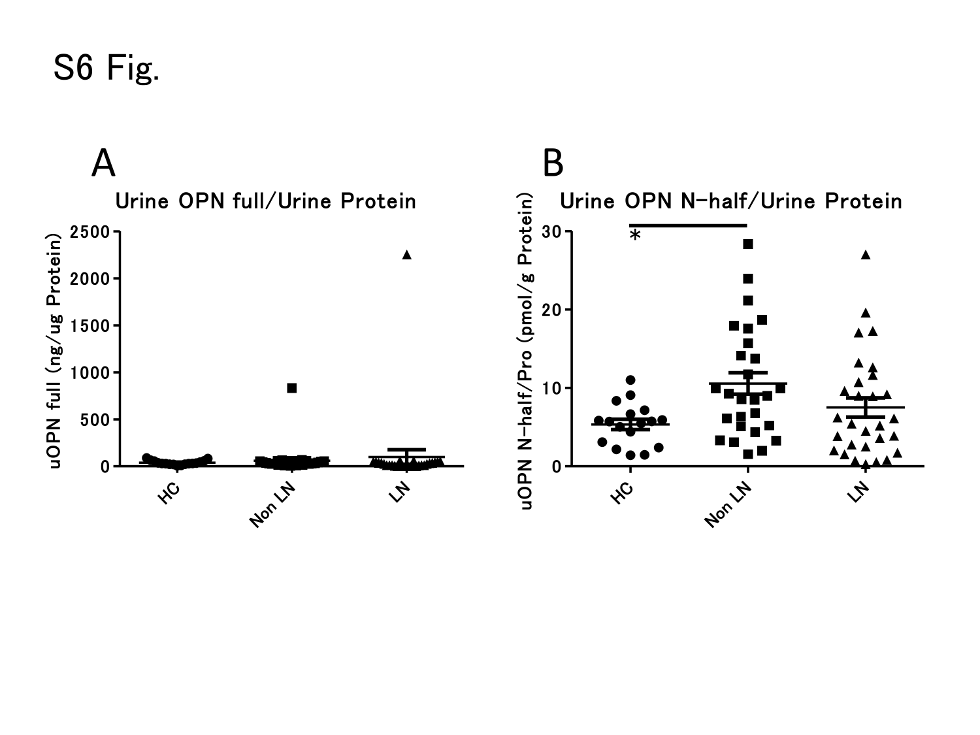

Supplement: S6 Fig — Concentration of urine OPN full (A) and urine OPN N-half (B) corrected by urine protein level in healthy controls (HC), SLE patients without LN (Non-LN), and those with LN. There was a significant difference in urine OPN N-half/urine protein ratio between HC and SLE patients (* p < 0.05 by Dunn’s test), however we did not find differences between SLE patients with and without LN. (TIF) [file pone.0167141.s006.tif]

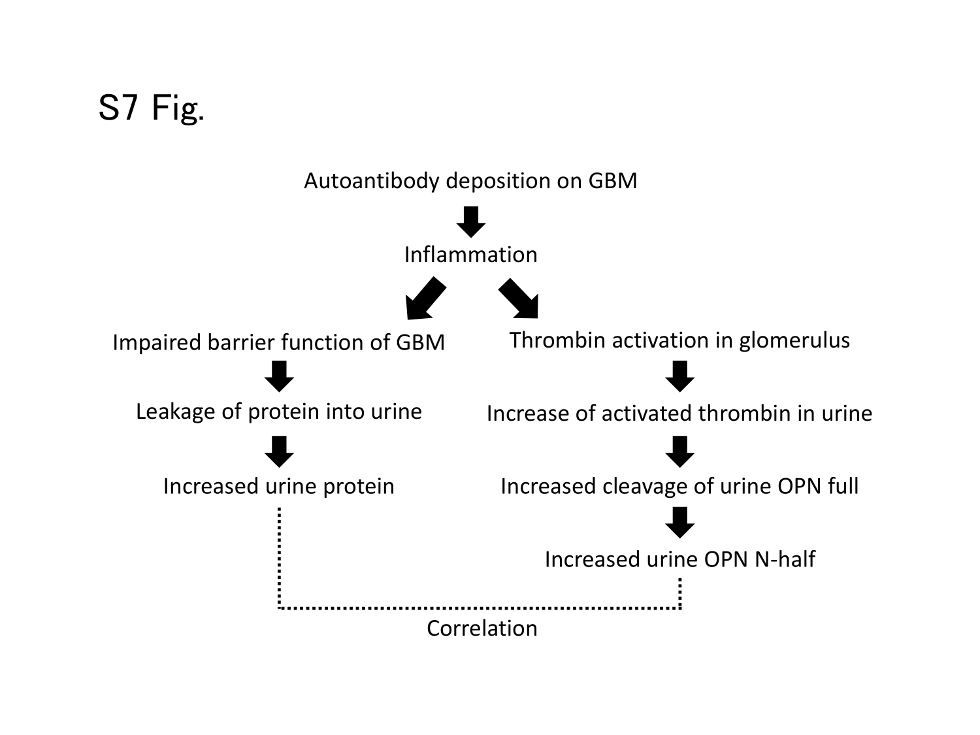

Supplement: S7 Fig — In active glomerulonephritis, the leakage of protein into urine is increased because of the impairment of glomerular barrier function as a result of inflammation around the GBM. Activation of thrombin is therefore increased locally at the same time. Activated thrombin can then cleave OPN in the urine. GBM: glomerular basement membrane. (TIF) [file pone.0167141.s007.tif]

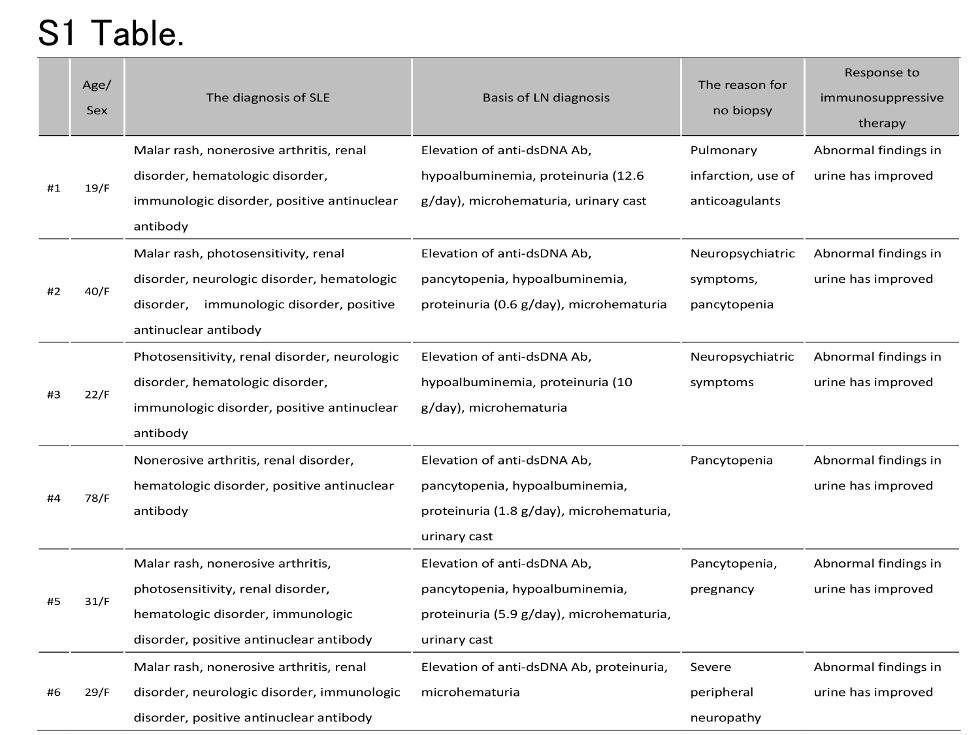

Supplement: S1 Table — All 6 patients fulfilled the criteria for SLE. Although we could not perform renal biopsy for compelling reasons, they were clinically diagnosed as having LN. (TIF) [file pone.0167141.s008.tif]
